# Efficacy and safety of posterior short-segment versus long-segment pedicle screws fixation for thoracolumbar burst fractures: A systematic review and meta-analysis

**DOI:** 10.1097/MD.0000000000042699

**Published:** 2025-06-06

**Authors:** Dandan Yu, Yuxuan Zhang, Xia Li, Wei Wang, Zengming Li, Jun Xiao

**Affiliations:** a Guangzhou Special Service Recuperation Center of PLA Rocket Force, Guangzhou, Guangdong, People’s Republic of China.

**Keywords:** analysis, long, meta, segment fixation, segment fixation, short, thoracolumbar burst fractures

## Abstract

**Background:**

To evaluate the effectiveness and safety of fixation levels with pedicle screw fixation for thoracolumbar burst fractures (TLBF).

**Methods:**

A systematic and comprehensive literature search was performed from inception to May 2024 in both English and Chinese databases, involving Medline, Cochrane Library, Embase, China National Knowledge Infrastructure Database, Wanfang Database, Chongqing VIP information, and SinoMed. Clinical trials of short-segment fixation and long-segment fixation (LSF) in the treatment of thoracolumbar burst fractures were included. Quality of included trials were assessed according to the methodological index for non-randomized studies (MINORS). Data analysis was conducted by using Review Manager 5.4 software and Stata. The quality of evidence in this systematic review was evaluated using the GRADE evidence quality evaluation system.

**Results:**

Seventeen eligible trials with a total of 1031 patients were included in this meta-analysis. Meta-analysis revealed that intraoperative bleeding (MD = −36.64, 95% CI = −56.36 to −16.92, *Z* = 3.64, *P* = .0003) and operation time (MD = −25.73, 95% CI = −46.56 to −4.90, *Z* = 2.42, *P* = .02) in the LSF group were higher than those in the short-segment fixation group. There were no significant differences in terms of the final follow-up sagittal index (MD = 1.64, 95% CI = −0.75 to 4.03, *Z* = 1.35, *P* = .18) and the final follow-up Oswestry disability index (MD = −2.94, 95% CI = −9.74 to 3.85, *Z* = 0.85, *P* = .40) between the 2 groups. The LSF group had the advantages of better the final follow-up Cobb angle (MD = 2.52, 95% CI = 0.35–4.70, *Z* = 2.27, *P* = .02), the final follow-up visual analog scale (MD = 0.09, 95% CI = 0.04–0.14, *Z* = 3.59, *P* = .0003) and lower the final follow-up implant failure (MD = 3.43, 95% CI = 1.78–6.62, *Z* = 3.69, *P* = .0002). The funnel plots and Egger test showed some evidence of asymmetry, suggesting publication bias or small sample effect was existed.

**Conclusion:**

For thoracolumbar burst fractures, LSF can better improve patients’ low back pain and better maintain postoperative orthopedic effect.

## 1. Introduction

Thoracolumbar fractures of the spine are the most common site of traumatic spinal fractures due to significant biomechanical stress at the junction between the mobile lumbar spine and the semirigid thoracic spine,^[1]^ and it is estimated that about 90% of spinal fractures occur at the thoracolumbar level.^[2,3]^ Due to high-energy trauma, approximately 10% to 20% of thoracolumbar fractures are burst fractures.^[4]^ Based on the 3-column model, burst fractures are the result of mechanical failure of the front and middle columns under pressure. The most important feature is the potential destruction of the posterior wall of the vertebral body, followed by the push of bone fragments backward into the spinal canal, which can lead to nerve damage^[5]^ and associated symptoms with the progression of kyphotic deformity.^[6]^ Therefore, for the treatment of TLBF with surgical indications, it is recommended to reestablish spinal stability, correct kyphotic deformity, and effectively decompress the spinal cord and nerves.^[6,7]^

The posterior pedicle screw fixation system is considered to be an effective method for the treatment of TLBF.^[8,9]^ However, the fixation segment of TLBF has been controversial.^[10–15]^ long-segment fixation (LSF) has the advantages of better fixed strength and dispersed stress, reducing the possibility of collapse of injured vertebra and instrument failure. However, LSF result in more surgical trauma, sacrifice more spinal motion segments, and accelerate adjacent disc degeneration.^[16,17]^ SSF fixation has the advantages of less invasive surgery, preservation of active segments and reduction of stress in adjacent segments, but the incidence of kyphotic deformity progression and instrument failure is higher.^[18–22]^ The only meta-analysis comparing TLBF with short and LSF was published in 2016,^[23]^ but we have reservations about the results because the included papers included reviews. Subsequently, a series of clinical studies^[8,9,24]^ compared the clinical and radiological effects of posterior SSF versus posterior LSF, but the results remain controversial. Considering that the controversy surrounding this topic has continued for more than a decade without resolution, analysis of controlled clinical trials incorporating both short and long segmental fixation in the treatment of TLBF is warranted.

## 2. Materials and methods

### 2.1. Search strategy

Electronic databases including Medline, Cochrane Library, Embase, China National Knowledge Infrastructure Database, Wanfang Database, Chongqing VIP information, and SinoMed were systematically searched for eligible studies from inception up to May 2024. Papers were retrieved using the title and keywords in the abstract: “thoracolumbar burst fractures” “fixation.” For PubMed, as an example, the retrieval formula was: thoracolumbar burst fractures [Title/Abstract]) AND (fixation [Title/Abstract]). In addition, the relevant conference papers, degree papers and other gray literature were also in the scope of the search. The literature search process is shown in the flow chart.

### 2.2. Selection criteria

Trials with the following characteristics were included: population: patients with confirmed pathological thoracolumbar burst fractures based on computed tomography and plain radiographs. Intervention/ Control: comparison of the SSF and LSF of surgical management. Outcome: at least one of the following outcomes were reported: intraoperative bleeding, operation time, the final follow-up Cobb angle, the final follow-up sagittal index, the final follow-up visual analog scale (VAS), the final follow-up Oswestry disability index (ODI), the final follow-up implant failure. Study design: clinical controlled trial, including retrospective cohort study, case-control study. We excluded studies that were duplicate reports of earlier trials or reports with incomplete or erroneous data and articles whose full text we were unable to acquire.

### 2.3. Data extraction

According to the preestablished screening criteria, 2 authors independently screened the literature, extracted the data and evaluated the quality and cross-checked them. Disagreements were resolved by consensus or consultation with the senior reviewer. A Excel data extraction table was established, which included literature sources, sample size, demographic baseline characteristics (including age, sex, etc), interventions, outcomes, and follow-up.

### 2.4. Quality assessment

Studies were graded independently using the methodological index for non-randomized studies (MINORS)^[25]^ by each reviewer. There were 12 evaluation indexes, each of which was divided into 0 to 2 points. A score of 0 indicates no relevant information was reported. A score of 1 indicates coverage but insufficient information. A score of 2 indicates that sufficient information was reported and provided.

### 2.5. Statistical analysis

Meta-analysis was performed using Review Manager 5.4 provided by the Cochrane Collaboration. Risk ratios were calculated for binary outcomes and mean difference for continuous outcomes, along with 95% confidence intervals (CI). Heterogeneity across studies was assessed using a standard Chi square test (statistical heterogeneity was considered significant at *P* > .1) and the *I*^2^ statistic.^[26]^ The fixed effect model was applied if *I*^2^ < 50% and *Q* test showed *P* > .1. The random effect model was adopted if *I*^2^ ≥ 50% and *Q* test show *P* ≤ .1. Publication bias tests using funnel plots were conducted in case where there were ≥ 10 included studies. Quantitative analysis of publication bias was performed using Egger test.

### 2.6. Quality of evidence GRADE assessment and recommendation grade

The quality of evidence in this systematic review was evaluated using the GRADE evidence quality evaluation system, which involves 5 evaluation items: risk of bias, inconsistency, indirectness, imprecision, and publication bias. The evidence level was divided into 4 grades: very low, low, moderate, and high.

## 3. Results

### 3.1. Search process

A total of 5311 papers were initially retrieved, and duplicate papers were checked and deleted by NoteExpress software (n = 3127). A total of 1771 documents were excluded after reading titles and abstracts according to the exclusion criteria, and 17 papers were finally included.

### 3.2. Characteristics of the included studies

A total of 17 studies^[12,14,24,27–40]^ were included, of which 1^[34]^ was split into 2 trials due to the different ways of grouping. The total sample size was 1031 cases. The maximum sample size was 160 cases and the minimum sample size was 12 cases. The detailed characteristics of the included studies are summarized in Table 1.

**Table 1:** Characteristics of the included studies.

References	Country	No. of patients		Surgical path	Gender (male/female)		Age (yr)		Follow-up time (mo)		Outcomes
		SSF	LSF	Anterior/posterior	SSF	LSF	SSF	LSF	SSF	LSF	
Yong et al^[27]^	China	78	26	Posterior	44/34	21/5	27–73 (50.4 ± 11.5)	23–75 (48.5 ± 13.8)	19.5 ± 7.0	20.8 ± 8.8	A B C E F G
Lei and Chenqiang^[28]^	China	40	42	Posterior	46/36		40.7 (21–60)		12		A B C E G
Qiang^[29]^	China	80	80	Posterior	–	–	18–60		36		A B
Xiaofeng and Feng^[30]^	China	50	50	Posterior	30/20	28/22	20–66 (44.3 ± 4.6)	21–67 (46.3 ± 3.9)	12		A B C E
Li^[31]^	China	20	20	Posterior	15/5	16/4	15–57	16–60	–	–	A B C G
Feng et al^[32]^	China	38	35	Posterior	28/10	28/7	18–55 (30.6 ± 5.2)	14–58 (35.6 ± 4.3)	14–32 (18.6 ± 3.6)		A B C E G
Ye et al^[33]^	China	6	6	Posterior	–	–	16–55 (32.6 ± 10.7)		10–34 (29.6 ± 9.1)		A B C G
Wu et al^[17]^	China	6	9	Posterior	–	–	56–78 (66.8 ± 9.2)		10–34 (29.6 ± 9.1)		A B C G
Choudhury et al^[24]^	Dhaka	40	40	Posterior	32/8	35/5	32.30 ± 11.85	33.13 ± 10.29	28.48 ± 3.735	29.50 ± 3.113	A B C G
Dobran et al^[14]^	Italy	30	30	Posterior	18/12	19/11	52.63 ± 19.43	49.23 ± 19.27	32.5 (18–58)	30.8 (18–58)	B C G
Canbek et al^[34]^	Turkey	10	15	Posterior	6/4	8/7	32.3 (17–52)	36 (19–50)	72.3 (31–102)	70.46 (5–104)	C D
Ugras et al^[35]^	Turkey	12	14	Posterior	6/6	6/8	34.33 ± 13.75	34.79 ± 16.79	20	28	C D E F G
Sapkas et al^[36]^	Greece	20	30	Posterior	12/8	20/10	33 (13–52)	35 (17–55)	34 (25–70)	36 (24–72)	C G
Uzumcugil et al^[37]^	Turkey	27	15	Posterior	18/9	10/5	39.3 ± 16.65	39.3 ± 15.51	28.41 ± 12.7	27.73 ± 10.23	C D E F G
Kim et al^[38]^	Korea	28	32	Posterior	16/12	22/10	41.0 ± 16.3	39.5 ± 13.5	21.0 ± 7.4		C D F G
Lee et al^[39]^	India	11	10	Posterior	7/4	7/3	45.7 ± 12.9		13.74 ± 5.58 (3–27)		C G
Altay et al^[12]^	Turkey	32	31	Posterior	19/13	18/13	42.6 ± 14.9	44.8 ± 14.9	36 (18–58)	33 (18–58)	D G
Tezeren and Kuru^[40]^	Turkey	9	9	Posterior	7/2	8/1	32 ± 13 (18–56)	34 ± 11 (17–53)	29 ± 5 (23–38)	29 ± 4 (24–40)	A B D G

A: intraoperative bleeding, B: operation time, C: the final follow-up Cobb angle, D: the final follow-up sagittal index, E: the final follow-up visual analog scale, F: the final follow-up ODI, G: the final follow-up implant failure.

LSF = long-segment fixation, ODI = Oswestry disability index, SSF = short-segment fixation.

### 3.3. Results of quality assessment

None of the included studies reported the following 3 items: inclusion of consecutive patients, unbiased assessment of the study endpoint, prospective calculation of the study size. All studies scored between 15 and 17. The risk of bias of the included studies is shown in Table 2.

**Table 2:** Methodological quality evaluation of the included studies.

MINORS items	Yong et al^[27]^	Lei and Chenqiang^[28]^	Qiang^[29]^	Xiaofeng and Feng^[30]^	Li^[31]^	Feng et al^[32]^	Ye et al^[33]^	Choudhury et al^[24]^	Dobran et al^[14]^	Canbek et al^[34]^	Ugras et al^[35]^	Sapkas et al^[36]^	Uzumcugil et al^[37]^	Kim et al^[38]^	Lee et al^[39]^	Altay et al^[^^12]^	Tezeren and Kuru^[40]^
1. A clearly stated aim	2	2	2	2	2	2	2	2	2	2	2	2	2	2	2	2	2
2. Inclusion of consecutive patients	0	0	0	0	0	0	0	0	0	0	0	0	0	0	0	0	0
3. Prospective collection of data	2	2	2	2	2	2	2	2	2	2	2	2	2	2	2	2	2
4. Endpoints appropriate to the aim of the study	2	2	1	2	2	2	2	2	1	1	2	1	2	2	1	1	1
5. Unbiased assessment of the study endpoint	0	0	0	0	0	0	0	0	0	0	0	0	0	0	0	0	0
6. Follow-up period appropriate to the aim of the study	2	2	2	2	0	2	2	2	2	2	2	2	2	2	2	2	2
7. Loss to follow up <5%	2	2	2	2	2	2	2	0	1	1	1	1	2	2	2	1	1
8. Prospective calculation of the study size	0	0	0	0	0	0	0	0	0	0	0	0	0	0	0	0	0
9. An adequate control group	1	1	1	1	1	1	1	1	1	1	1	1	1	1	1	1	1
10. Contemporary groups	2	2	2	2	2	2	2	2	2	2	2	2	2	2	2	2	2
11. Baseline equivalence of groups	2	2	2	2	2	2	2	2	2	2	2	2	2	2	2	2	2
12. Adequate statistical analyses	2	2	2	2	2	2	2	2	2	2	2	2	2	2	2	2	2
Total score	17	17	16	17	15	17	17	15	15	15	16	15	17	17	16	15	15

1: A score of 0 indicates unreported; 2: a score of 1 indicates coverage but insufficient information; 3: a score of 2 indicates that sufficient information was reported and provided.

MINORS = methodological index for non-randomized studies.

### 3.4. Results of the meta-analysis for outcomes

#### 3.4.1. *Intraoperative bleeding*

**Figure 1.**
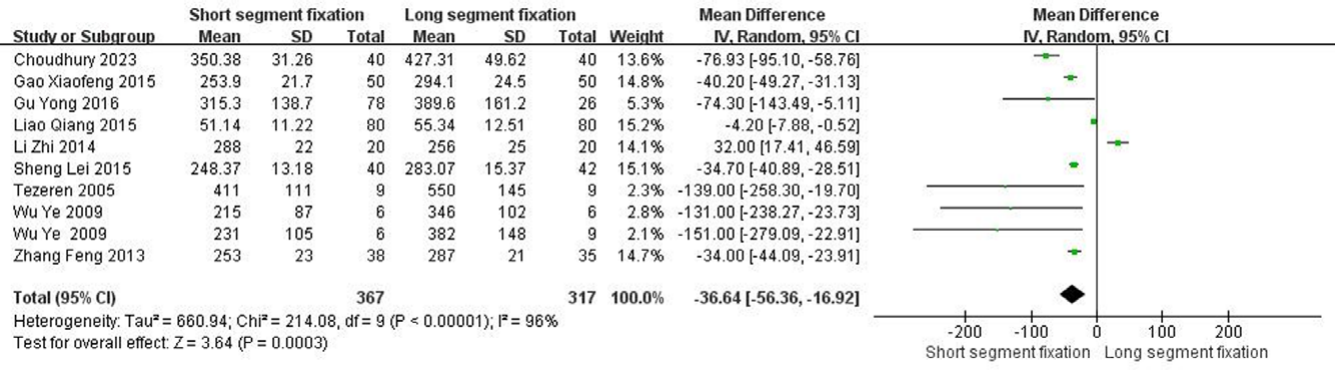
Forest plot of intraoperative bleeding between the 2 groups.

Nine articles including ten trials showed detailed comparisons of intraoperative bleeding. There was heterogeneity between the literature (Chi^2^ = 214.08, *I*^2^ = 96%, *P* < .00001), and a random effect model was selected for meta-analysis. The amount of intraoperative bleeding in the SSF group was less than that in the LSF group (MD = −36.64, 95% CI = −56.36 to −16.92, *Z* = 3.64, *P* = .0003) (Fig. 1) (units: milliliters).

#### 3.4.2. *Operation time*

**Figure 2.**
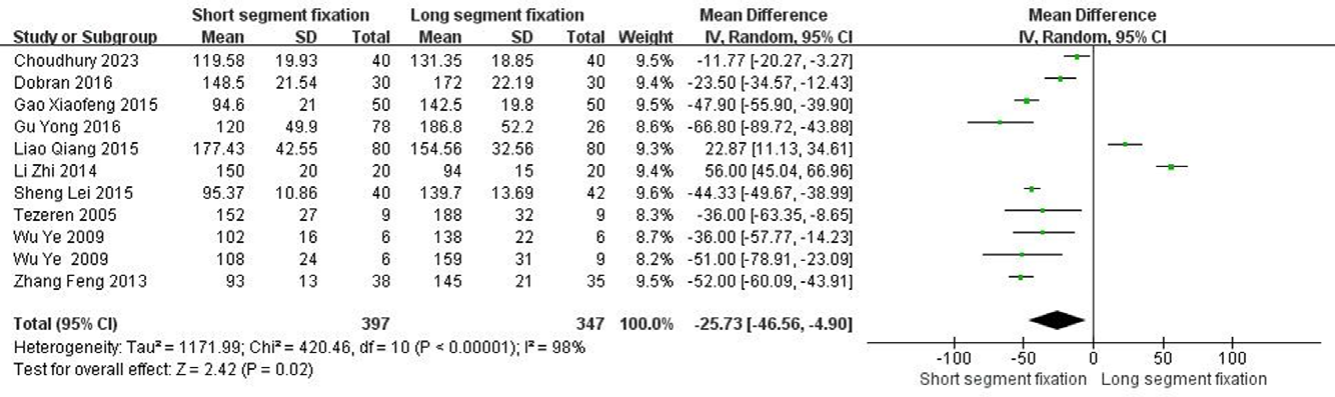
Forest plot of operation time between the 2 groups.

Ten articles including eleven trials showed detailed comparisons of the operation time. There was heterogeneity between the literature (Chi^2^ = 420.46, *I*^2^ = 98%, *P* < .00001), and a random effect model was selected for meta-analysis. The operation time of the SSF group was shorter than that of the LSF group (MD = −25.73, 95% CI = −46.56 to −4.90, *Z* = 2.42, *P* = .02) (Fig. 2) (units: minutes).

#### 3.4.3. The final follow-up Cobb angle

**Figure 3.**
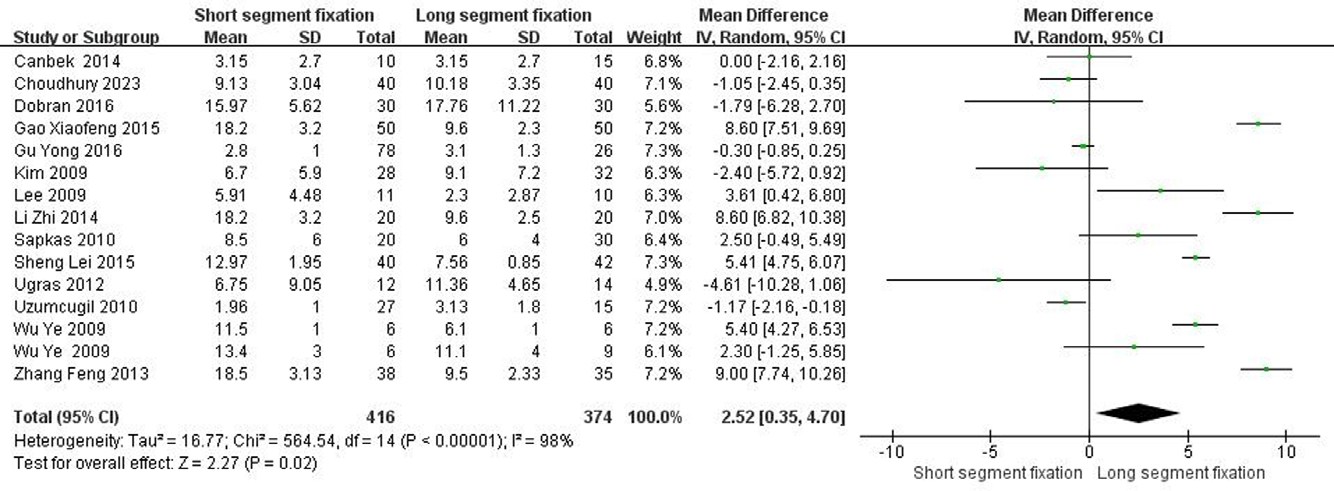
Forest plot of the final follow-up Cobb angle between the 2 groups.

Fourteen articles including fifteen trials showed detailed comparisons of the final follow-up Cobb angle. There was heterogeneity between the literature (Chi^2^ = 564.54, *I*^2^ = 98%, *P* < .00001), and a random effect model was selected for meta-analysis. The reduction of the final follow-up Cobb angle in the LSF group was more than that in the SSF group (MD = 2.52, 95% CI = 0.35–4.70, *Z* = 2.27, *P* = .02) (Fig. 3).

#### 3.4.4. The final follow-up sagittal index

**Figure 4.**
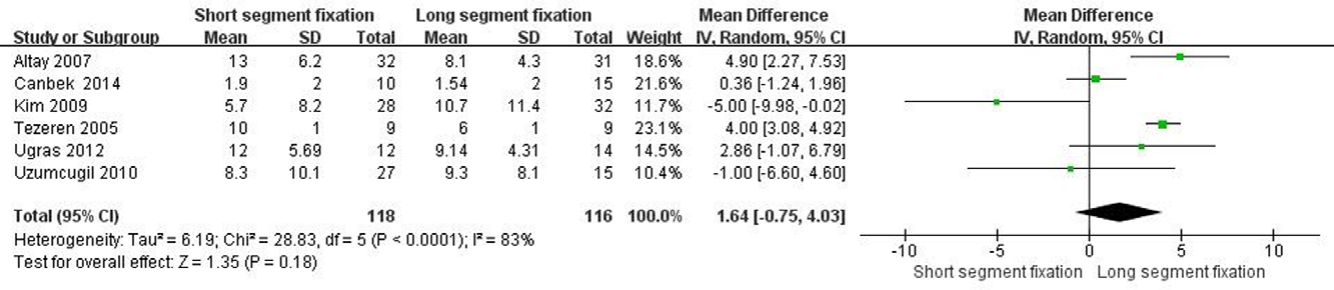
Forest plot of the final follow-up sagittal index between the 2 groups.

Six articles showed detailed comparisons of the final follow-up sagittal index. There was heterogeneity between the literature (Chi^2^ = 28.83, *I*^2^ = 83%, *P* < .0001), and a random effect model was selected for meta-analysis. The final follow-up sagittal index of the SSF and LSF groups were not significantly different (MD = 1.64, 95% CI = −0.75 to 4.03, *Z* = 1.35, *P* = .18) (Fig. 4).

#### 3.4.5. The final follow-up visual analog scale

**Figure 5.**
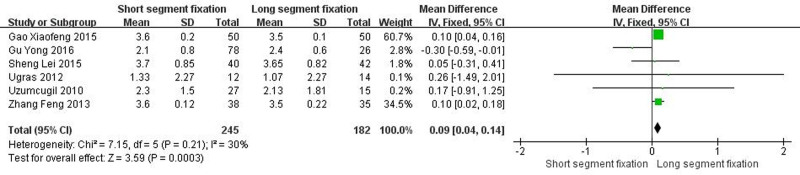
Forest plot of the final follow-up VAS between the 2 groups. VAS = visual analog scale.

Six articles showed detailed comparisons of the final follow-up VAS. There was heterogeneity between the literature (Chi^2^ = 7.15, *I*^2^ = 30%, *P* = .21), and a fixed effect model was selected for meta-analysis. The reduction of the final follow-up VAS in the LSF group was more than that in the SSF group (MD = 0.09, 95% CI = 0.04–0.14, *Z* = 3.59, *P* = .0003) (Fig. 5).

#### 3.4.6. The final follow-up oswestry disability index

**Figure 6.**
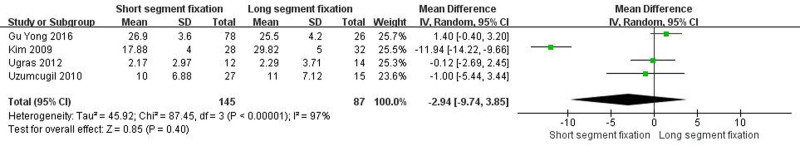
Forest plot of the final follow-up ODI between the 2 groups. ODI = Oswestry disability index.

Four articles showed detailed comparisons of the final follow-up ODI. There was heterogeneity between the literature (Chi^2^ = 87.45, *I*^2^ = 97%, *P* < .00001), and a random effect model was selected for meta-analysis. The final follow-up ODI of the SSF and LSF groups were not significantly different (MD = −2.94, 95% CI = −9.74 to 3.85, *Z* = 0.85, *P* = .40) (Fig. 6).

#### 3.4.7. The final follow-up implant failure

**Figure 7.**
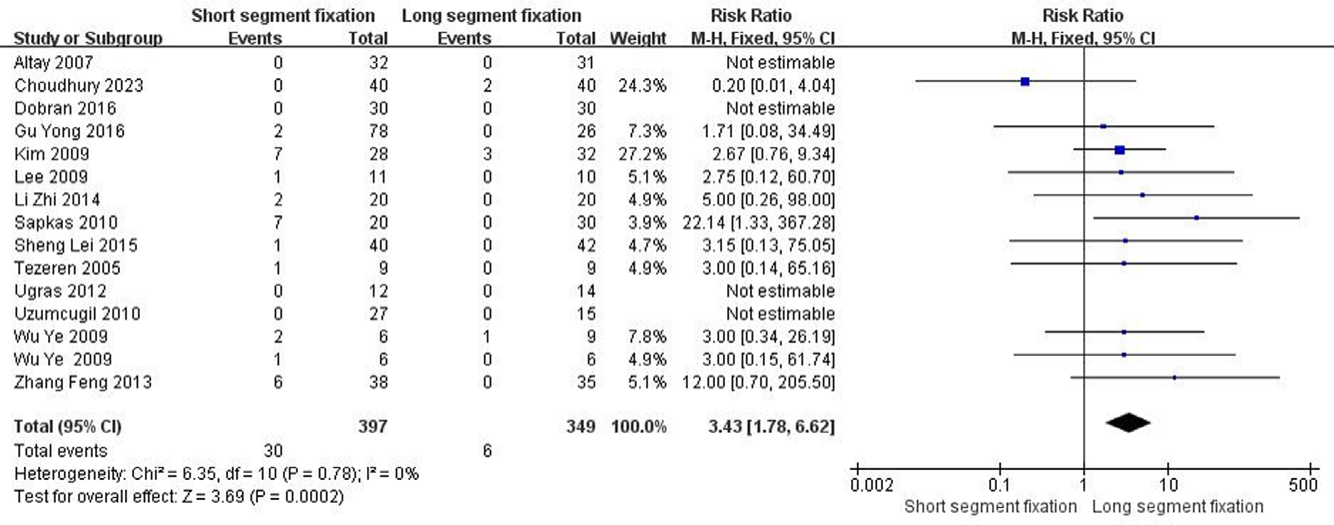
Forest plot of the final follow-up implant failure between the 2 groups.

Fourteen articles including fifteen trials showed detailed comparisons of the final follow-up implant failure. There was heterogeneity between the literature (Chi^2^ = 6.35, *I*^2^ = 0%, *P* = .78), and a fixed effect model was selected for meta-analysis. The incidence of the final follow-up implant failure in the LSF group was less than that in the SSF group (MD = 3.43, 95% CI = 1.78–6.62, *Z* = 3.69, *P* = .0002) (Fig. 7).

#### 3.4.8. Publication bias

**Figure 8.**
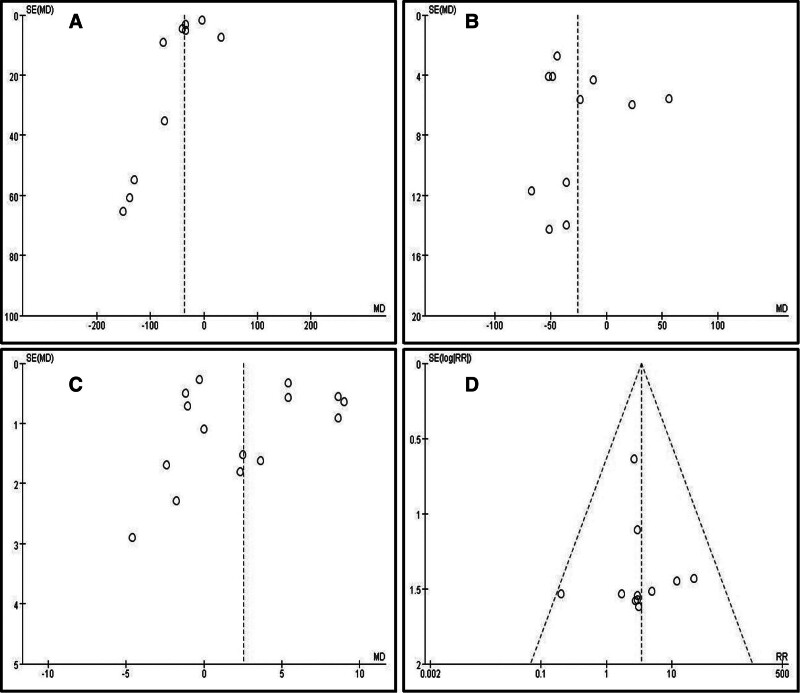
Funnel plot of publication bias: (A) intraoperative bleeding; (B) operation time; (C) the final follow-up Cobb angle; (D) the final follow-up implant failure.

**Figure 9.**
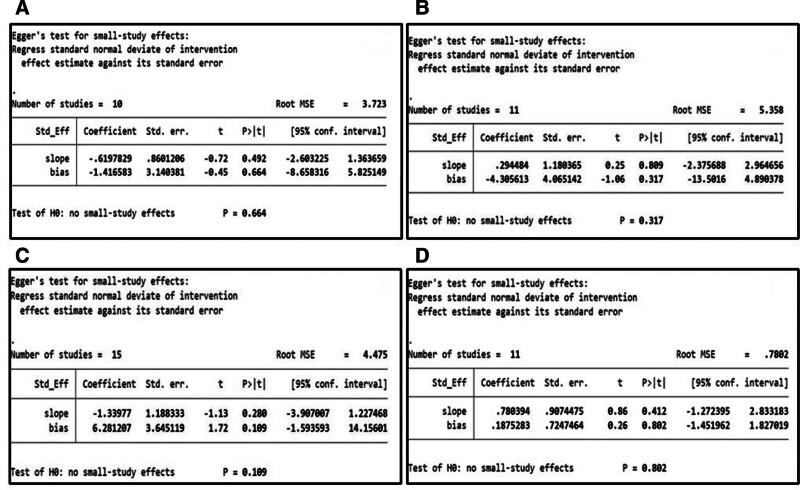
Egger test of publication bias. (A) intraoperative bleeding (*P* = .664 > .05); (B) operation time (*P* = .317 > .05); (C) the final follow-up Cobb angle (*P* = .109 > .05); (D) the final follow-up implant failure (*P* = .802 > .05).

Publication bias was evaluated by visually inspecting funnel plots when at least 10 studies were included in the meta-analysis. We further used Egger test to comprehensively analyze the publication bias of the article. Egger test is a method of quantitative analysis of publication bias. The publication bias was significantly existed if *P* < .05. Therefore, 4 funnel plots were produced for the outcomes of intraoperative bleeding, operation time, the final follow-up Cobb angle and the final follow-up implant failure (Fig. 8). The Egger test showed that the publication bias was not significantly existed (Fig. 9). The funnel plots showed some evidence of asymmetry, suggesting publication bias or small sample effect was probably existed.

#### 3.4.9. GRADE assessment of evidence quality

According to the GRADE standard, the final follow-up implant failure was rated as low quality evidence. The other 6 outcomes were rated as very low quality evidence. The evidence quality grading of outcome is shown in Table 3.

**Table 3:** The evidence quality grading of outcome.

Outcome	Number of trials	Quality assessment of evidence					Sample size/case		Quality grading
		risk of bias	inconsistency	indirectness	imprecision	publication bias	SSF	LSF	
Intraoperative bleeding	10	Downgrade 1 level^1^	Downgrade 1 level^2^	No downgrade	No downgrade	Downgrade 1 level^4^	367	317	Very low
Operation time	11	Downgrade 1 level^1^	Downgrade 1 level^2^	No downgrade	No downgrade	Downgrade 1 level^4^	397	347	Very low
The final follow-up Cobb angle	15	Downgrade 1 level^1^	Downgrade 1 level^2^	No downgrade	No downgrade	Downgrade 1 level^4^	416	374	Very low
The final follow-up sagittal index	6	Downgrade 1 level^1^	Downgrade 1 level^2^	No downgrade	Downgrade 2 level^3^	Downgrade 2 level^4^	118	116	Very low
The final follow-up visual analog scale	6	Downgrade 1 level^1^	No downgrade	No downgrade	No downgrade	Downgrade 2 level^4^	245	182	Very low
The final follow-up Oswestry disability index	4	Downgrade 1 level^1^	Downgrade 1 level^2^	No downgrade	Downgrade 2 level^3^	Downgrade 2 level^4^	145	87	Very low
The final follow-up implant failure	14	Downgrade 1 level^1^	No downgrade	No downgrade	No downgrade	Downgrade 1 level^4^	397	349	Low

Downgrade 1 level^1^ indicates that the studies included in the review have defects in the following aspects: inclusion of consecutive patients, unbiased assessment of the study endpoint, prospective calculation of the study size; downgrade 1 level^2^ indicates heterogeneity test, *I*^2^ > 50%; downgrade 2 level^3^ indicates that confidence interval spans the line of no effect and the sample size is small; downgrade 1 level^4^ indicates publication bias; downgrade 2 level^4^ indicates that it is uncertain whether there is publication bias.

LSF = long-segment fixation, SSF = short-segment fixation.

## 4. Discussion

Thoracolumbar burst fractures are the most common traumatic spinal fractures caused by axial load, with or without buckling force, affecting the anterior and middle columns of the vertebral body.^[41]^ Due to the high-energy trauma and the special biomechanical effects of the thoracolumbar, surgery is often required.^[42]^ Posterior pedicle screw fixation is one of the most common and simplest procedures for thoracolumbar burst fractures^[1]^. However, the surgical fixation of segments has long been controversial.^[10–15]^

Girardo et al^[14,43,44]^ noted in their study that there was no statistically significant difference between long and short-segment fixation in improving the clinical symptoms and correcting kyphosis of thoracolateral burst fractures. Sharif et al^[1]^ also recommend that for thoracolumbar junction blowout fractures, the combination of fracture horizontal screws is preferred to increase structural strength, and if fracture horizontal screws are not available, LSF should be used. Chokshi et al^[45]^ showed that the inclusion of fracture level (load sharing core, LSC ≤ 6) in SSF of thoracolumbar fractures resulted in good kyphotic correction and correction maintenance. Lee et al^[46,47]^ also reported a significant difference in the loss of correction Angle with SSF, and indicated that short posterior internal fixation was insufficient in cases with a LSC score of 7 or more. Jindal et al^[21]^ suggest that SSF is adequate for almost all type A and type B fractures of the non-tetanic spine. There may be a slight recurrence of kyphosis, but the clinical impact is minimal. Martiniani et al^[48]^ argue that it is critical to assess a patient’s overall sagittal alignment. SSF can correct focal malformations, while LSF is required for kyphosis with global sagittal imbalance. All of these studies were small prospective trials or retrospective analyses, with a major limitation being the lack of primary evidence.

From the patient’s point of view, pain relief and kyphotic deformity correction are the main indicators of surgical management of TLBF.^[49]^ In this systematic review, both methods significantly improved postoperative VAS score and ODI score of patients. Surprisingly, the improvement of VAS score was better than that of SSF despite the greater trauma of LSF (Fig. 5, *P* < .05). We speculated that the reason was that LSF could better correct kyphosis, and the maintenance of kyphosis was better than SSF (Fig. 3, *P* < .05). It is well known that although thoracolumbar deformity is not associated with thoracolumbar pain,^[50–54]^ secondary lumbar lordosis is significantly associated with pain.^[55,56]^ In addition, when the local kyphosis increases, the length and tension of the paraspinous muscles increase due to the higher tension of the posterior tension band, which in turn causes pain.^[6]^ Interestingly, although LSF was superior to SSF in maintaining kyphotic deformity, there was no statistical difference in maintaining the overall sagittal balance of the spine (Fig. 4, *P* > .05). The main reason may be a certain degree of kyphosis, the spine can be compensated by lumbar lordosis and pelvic backward tilt, so as to maintain the overall sagittal balance of the spine. Overall, for TLBF, LSF is better than SSF in improving some clinical efficacy and radiological indicators, but it should be noted that LSF has shortcomings such as long operation time and large amount of blood loss (Fig. 1, *P* < .05; Fig. 2, *P* < .05), which may lead to an increased risk of implant infection.

This study has some limitations. First of all, among the 17 studies included, there are few high-quality clinical control research articles and lack of prospective research, which inevitably leads to publication bias and selection bias, affecting the reliability and authenticity of the research results. Second, there are differences in the age, disease stage and follow-up times of the included patients, resulting in the presence of clinical heterogeneity, which is also an important factor affecting the results of the study. Third, it is difficult to grasp the details of the implementation of each study, such as whether the patient assisted bone grafting, whether there was a combination of osteoporosis, whether the injured vertebra is fixed and the transverse connection is used, the doctor’s proficiency, and the authenticity and integrity of the medical record. Finally, we performed a meta-analysis that provided indirect estimates of the evidence. Therefore, it is not a substitute for evidence in real-world research.

## 5. Conclusion

The results of this meta-analysis show that in the surgical treatment of TLBF, LSF is superior to SSF in improving thoracolumbar pain and reducing postoperative kyphotic loss, and has a lower risk of implant failure.

## Author contributions

**Conceptualization:** Dandan Yu, Jun Xiao.

**Data curation:** Yuxuan Zhang, Wei Wang.

**Investigation:** Yuxuan Zhang, Xia Li.

**Writing – original draft:** Dandan Yu, Xia Li, Zengming Li, Jun Xiao.

**Writing – review & editing:** Dandan Yu.
